# Radical cystectomy in patients aged < 80 years versus ≥ 80 years: analysis of preoperative geriatric assessment scores in predicting postoperative morbidity and mortality

**DOI:** 10.1007/s00345-024-05248-y

**Published:** 2024-09-30

**Authors:** Gregor Duwe, Isabel Wagner, Katarzyna E. Banasiewicz, Lisa Johanna Frey, Nikita Dhruva Fischer, Johann Bierlein, Niklas Rölz, Maximilian Haack, Rene Mager, Christopher C. M. Neumann, Katharina Boehm, Peter Sparwasser, Igor Tsaur, Mohamed M. Kamal, Axel Haferkamp, Maximilian Peter Brandt, Thomas Höfner

**Affiliations:** 1 Department of Urology and Pediatric Urology, University Medical Center of the Johannes Gutenberg-University Mainz, Langenbeckstrasse 1, 55131 Mainz, Germany; 2 Department of Hematology, Oncology, and Cancer Immunology (CCM), Charité - Universitaetsmedizin Berlin, 10117 Berlin, Germany; 3 Department of Urology, University Hospital Carl Gustav-Carus, TU Dresden, 01307 Dresden, Germany; 4 Department of Urology, Faculty of Medicine, University Hospital, Eberhard Karls University, 72076 Tübingen, Tuebingen, Germany; 5 Department of Urology, Ordensklinikum Linz Elisabethinen, Fadinger Strasse 1, Linz, 4020 Austria

**Keywords:** Radical cystectomy, Geriatric assessment, Comorbidities, Postoperative complications, Morbidity, Mortality, Outcome improvement

## Abstract

**Purpose:**

Pre-operative assessment of surgical risk is essential for patient counselling in the elderly patient population. Our purpose was to compare validated geriatric assessment scores (GAS) in predicting postoperative morbidity and mortality in patients ≥ 80 years.

**Methods:**

Overall, eight preoperative GAS were assessed for each patient who received RC from 2016 to 2021. Postoperative morbidity was recorded according to the Clavien-Dindo classification (CDC) of surgical complications. Binary logistic regression analyses were used to determine prediction of 30-d morbidity and 90-d mortality in patients ≥ 80 years.

**Results:**

In total, 424 patients were analysed (77.4% male) with median age of 71 years (IQR: 68.82;70.69), of which 67 (15.8%) were ≥ 80 years. Patients age ≥ 80 years showed more 30-d CDC grade ≥ IIIb (41.07% vs. 27.74% compared to < 80 years, *p* < .001) and worse 90-d mortality (26.87% vs. 4.76%, *p* < .001). In patients ≥ 80 years, morbidity was predicted by simplified Frailty Index (sFI)  ≥ 2 (OR: 2.06, 95% CI: 1.27–3.34, *p* = .004), Eastern Cooperative Oncology Group (ECOG) performance status ≥ 2 (OR: 2.78, 95% CI: 1.18–6.54, *p* = .019) and severe Adult Comorbidity Evaluation (ACE)-27 score (OR: 2.07, 95% CI: 1.13–3.79, *p* = .019), while 90-d mortality was predicted by CDC grade ≥ IIIb (OR: 22.91, 95% CI: 8.74–60.09, *p* < .001) and ECOG ≥ 2 (OR: 2.87, 95% CI: 1.05–7.86, *p* = .04).

**Conclusion:**

Even in a high-volume center of RC, 90-d mortality is significantly higher in patients age ≥ 80. Our results suggest in patient age ≥ 80, sFI ≥ 2, ECOG performance status ≥ 2 and severe ACE-27 score as clinical cut-off value to evaluate alternative bladder-sparing concepts.

**Supplementary Information:**

The online version contains supplementary material available at 10.1007/s00345-024-05248-y.

## Introduction

Radical cystectomy (RC) is a valid treatment option for BCG (Bacillus Calmette-Guerin) unresponsive high-risk non-muscle-invasive bladder cancer and gold-standard for non-metastasized muscle-invasive bladder (MIBC) [1]. RC is considered as one of the most complex surgical procedures in urology. Due to cancer prevention and screening programs and world-wide demographic changes, a significant increase in median age of patients including fragile patients above 80 years undergoing RC is reported [2]. Despite recent improvements in perioperative and surgical management based on Enhanced Recovery after Surgery (ERAS^®^) pathways and robot-assisted surgery [3, 4], RC is still accompanied with high rates of short-term complications rates and 30-days (-d) mortality ranges between 1% and 3% [5]. Notably, these rates are significantly elevated with 30-d mortality up to 10% in patients 80 years and over [6, 7]. Moreover, increased postoperative complications are associated with overall mortality [8]. These circumstances make “bladder cancer the most expensive cancer, per patient, to care for and to treat” [9, 10]. The importance of hospital and surgeon volume as major determinants of morbidity and mortality is proven and internationally acknowledged [11], leading to current European Association of Urology (EAU) recommendations of at least 10, and preferably > 20, RCs performed annually [12]. European Association of Urology (EAU) guidelines for MIBC already strongly recommend to base treatment decision, whether to opt for radical cystectomy or on bladder-sparing treatments in “older/ frail patients” on a standardized geriatric assessment (GA) [12]. Finally, EAU guidelines only recommend the Charlson Comorbidity Index (CCI) as an example of usage due to the highest level of evidence to predict peri-operative, overall and cancer-specific mortality [13, 14]. Until now, there is low evidence with heterogeneous evaluations in GAS ability to predict postoperative complications, in particular in the elderly population [15, 16]. Thus, EAU guidelines are only able to conclude that chronological age alone should not be used to evaluate RC for elderly patients. Since number and nature of complications has a proven significant impact on perioperative mortality after RC, we see a crucial knowledge gap in the evidence of GAS to better predict perioperative complications in addition to mortality [8]. Thus, we sought to compare the most common GAS and the ability to predict severe postoperative complications rates in patients ≥ 80 years who underwent RC. Furthermore, we aimed to investigate a “threshold” limit for the best GAS that could be easily applied in daily clinical decision making when evaluating RC for the increasing number of octogenarian patients.

## Patients and methods

### Patient population and outcomes

We identified 429 patients who underwent RC by eight different surgeons at our institution from January 2016 to December 2021 through a clinical case database. The ethics committee of the Medical Association of Rhineland-Palatinate, Germany, approved the study (2021–15768). Inclusion criteria were age > 18 years and performed RC for either high-risk non-muscle invasive BC, muscle-invasive BC and, in rare cases, also in patients in palliative disease or non-bladder cancer pathologies. All patients underwent extended pelvic lymphadenectomy as part of institutional standard. Radical cystectomy and urinary diversion were performed by open or, since 2016, also robot-assisted approach per patient and surgeon preference. Since June 2019, we implemented the ERAS^®^ protocol, including various aspects in peri- and postoperative procedures, e.g., pain management and early mobilization. After discharge, most patients were transferred to an inpatient rehabilitation clinic for early recovery.

We extracted all available clinicopathologic and perioperative patient characteristics, including the American Society of Anaesthesiologists (ASA) risk stratification and Eastern Cooperative Oncology Group (ECOG) performance status. Furthermore, the following GAS were retrospectively calculated based on the available patients’ characteristics: CCI [17], Adult Comorbidity Evaluation (ACE)-*27* score [18], Preoperative Score to Predict Postoperative Mortality (POSPOM) [19], Identification of Seniors at Risk (ISAR) Screening [20] and the simplified Frailty Index (sFI) [16]. All categorical variables of the GAS were transformed into binary variables using the median split. Eventually, in-hospital complications were graded by the Clavien-Dindo Classification (CDC) of surgical complications and transformed into binary variables with CDC IIIb as clinically relevant severe complications based on previous literature [16, 21]. Furthermore, patients were stratified by age ≥ 80 to assess outcome paraments for geriatric patients.

### Statistical analysis

Statistical analysis was performed using IBM SPSS Statistics Version 27 (Armonk, NY: IBM Corp.). Continuous variables are presented as mean ± standard deviation (SD) or medians ± interquartile range (IQR) in accordance with the data distribution. Clinicopathologic and perioperative patient characteristics including GAS were stratified by CDC grade ≥ IIIb and patients age ≥ 80 years and tested for statistical significance by the Pearson x^2^ test or Mann Whitney U Test. A univariate and multivariate logistic regression model was applied to compare age with BMI and GAS in prediction of postoperative morbidity and mortality. All tests were 2-tailed and a *p-*value < 0.05 was considered statistically significant.

## Results

### Patient baseline characteristics and geriatric assessment scores

We identified 424 patients (77.4% males and 22.6% females) who underwent RC from 2016 to 2021 (Supplementary Table 1). Median age at time of RC was 71.00 years (68.82;70.68) and 67 (15.8%) patients were ≥ 80 years old at time of RC. Open surgery was performed in 380 patients (89.6%), while robotic-assisted surgery was performed in 44 patients (10.4%). Next, we have stratified patients with < 80 years and ≥ 80 years of age according to clinicopathologic and perioperative patient characteristics (Table 1). Patients ≥ 80 years of age were predominantly male (73.1%) and received incontinent urinary diversions only, compared to patients < 80 years with 18.7% of continent urinary diversions. While no statistically significant differences were observed in T- and N-Stage distribution, positive resection margin (R+) was more frequent in patients ≥ 80 years compared to < 80 years (20.69% vs. 9.48%, *p* = .046). Regarding GAS, we identified statistically significant differences in patients ≥ 80 years compared to < 80 years in the stratified POSPOM score ≥ 28 (28.4% vs. 0%, *p* < .001), ASA Classification ≥ 3 (72.3% vs. 58%) and CDC grade ≥ IIIb (41.1% vs. 27.7%). Moreover, patients ≥ 80 years had significant worse 30-d (16.4% vs. 1.7%) as well as 90-d (26.9% vs. 4.8%) postoperative mortality rates.

**Table 1:** Correlation of geriatric patients aged < 80 years versus ≥ 80 years with clinicopathologic characteristics, geriatric assessment scores and postoperative outcomes

Characteristic	Patients age < 80 years (%; SD), *n* = 357	Patients age ≥ 80 years (%; SD), *n* = 67	*p*-value
**Gender**			0.368
Male	279 (78.15)	49 (73.13)	
Female	78 (21.85)	18 (26.87)	
**Neoadjuvant Chemotherapy**			
None	329 (92.16)	66 (98.51)	0.059
Yes	28 (7.84)	1 (1.49)	
**RC Indication**			
Curative (bladder cancer)	333 (93.29)	63 (94.03)	0.82
Other (palliative, non-oncological)	24 (6.72)	4 (5.97)	
**Robotic-assisted RC**			
None	318 (89.08)	62 (92.54)	0.394
Yes	39 (10.92)	5 (7.46)	
**Urinary Diversion**			< 0.001*
Incontinent	287 (81.3)	65 (100)	
Continent	66 (18.7)	0 (0)	
**T-Stage**			0.851
pT < 2	101 (31.56)	20 (32.79)	
pT ≥ 2	219 (68.44)	41 (61.21)	
**N-Stage (lmyphatic spread)**			0.706
N0	213 (68.05)	37 (62.71)	
N+ (positive)	75 (23.96)	16 (27.12)	
NX	25 (7.99)	6 (10.17)	
**R-Stage (Surgical Margin)**			0.046*
R0	270 (88.24)	45 (77.59)	
R+ (positive)	29 (9.48)	12 (20.69)	
RX	7 (2.29)	1 (1.72)	
**ISAR-Screening**			0.014*
Mean (SD)	1.62 (SD: 0.92)	2.01 (1.16)	
**POSPOM (mean)**			< 0.001*
< 27	188 (100)	169 (71.61)	
≥ 28	0 (0)	67 (28.39)	
**ASA Classification**			0.03*
< 3	147 (42.00)	18 (27.69)	
≥ 3	203 (58.00)	47 (72.31)	
**ACE-27 Score**			0.135
None	48 (13.45)	4 (5.97)	
Mild	110 (30.81)	21 (31.34)	
Moderate	112 (31.37)	21 (31.34)	
Severe	87 (24.37)	21 (31.34)	
**Simplified Frailty Index**			0.097
< 2	235 (65.83)	37 (55.22)	
≥ 2	122 (34.17)	30 (44.78)	
**Clavien-Dindo Classification**			0.047*
< IIIb	198 (72.26)	33 (58.93)	
≥ IIIb	76 (27.74)	23 (41.07)	
**Survival 30 days after RC**			< 0.001*
Yes	351 (98.32)	56 (83.58)	
Died	6 (1.68)	11 (16.42)	
**Survival 90 days after RC**			< 0.001*
Yes	340 (95.24)	49 (73.13)	
Died	17 (4.76)	18 (26.87)	

Abbreviations: *statistically significant results, RC: Radical Cystectomy, ISAR: Identification of Seniors at Risk, POSPOM: Preoperative Score to Predict Postoperative Mortality, ASA: American Society of Anaesthesiologists risk stratification, ACE 27: Adult Comorbidity Evaluation 27. ** few patients received no commonly classified urinary diversion, e.g. permanent nephrostomy

**Table 2:** Binary logistic regression analysis of patients aged ≥ 80 years in addition to geriatric assessment scores to predict 30d postoperative clavien-dindo complications ≥ IIIb

Groups	Age ≥ 80 + Scores	Multivariate Analysis			Univariate Analysis		
		OR	95% CI	*p*-value	OR	95% CI	*p*-value
1	**Age ≥ 80**	1.87	1.02–3.41	0.042 *	1.82	1-3.29	0.049*
	**BMI (metric)**	1.02	0.97–1.07	0.553	1.01	0.96–1.06	0.762
2	**Age ≥ 80**	2.16	1.19–3.92	0.011*	1.82	1-3.29	0.049*
	ECOG < 2 (reference)						
	**ECOG ≥ 2**	2.78	1.18–6.54	0.019*	2.99	1.33–6.73	0.008*
3	**Age ≥ 80**	2.29	1.27–4.12	0.006*	1.82	1-3.29	0.049*
	ASA < 3 (reference)						
	**ASA ≥ 3**	1.19	0.75–1.91	0.462	1.27	0.8–2.03	0.305
4	**Age ≥ 80**	1.7	0.93–3.11	0.087	1.82	1-3.29	0.049*
	sFI < 2 (reference)						
	**sFI ≥ 2**	2.06	1.27–3.34	0.004*	2.12	1.31–3.43	0.002
5	**Age ≥ 80**	1.41	0.74–2.68	0.294	1.82	1-3.29	0.049*
	POSPOM < 28 (reference)						
	**POSPOM ≥ 28**	1.71	1-2.92	0.051	1.89	1.15–3.11	0.012
6	**Age ≥ 80**	1.76	0.94–2.28	0.078	1.82	1-3.29	0.049*
	ACE 27 mild (reference)						
	ACE27 moderate	0.85	0.45–1.61	0.626	0.97	0.39–2.44	0.955
	**ACE 27 severe**	2.07	1.13–3.79	0.019*	2.34	0.95–5.75	0.063

Abbreviations: OR: Odds ratio (β), CI: confidence interval, *statistically significant results, BMI: Body Mass Index, ECOG: Eastern Cooperative Oncology Group (Performance Status), ASA: American Society of Anaesthesiologists risk stratification, sFI: Simplified Frailty Index, POSPOM: Preoperative Score to Predict Postoperative Mortality, ACE 27: Adult Comorbidity Evaluation 27

### Assessment and comparison of different geriatric assessments scores in predicting severe Clavien-Dindo complications and 90 days survival

Postoperative CDC ≥ IIIb occurred significantly more often in patients ≥ 80 years old (41.1% vs. 27.7%, *p* < .047), POSPOM score ≥ 28 (68.7% vs. 31.3%, *p* = .011), sFI ≥ 2 (40.2% vs. 24%, *p* = .002), CCI ≥ 5 (40.4% vs. 21.2% CCI 3–4 vs. 20.2% CCI 1–2, *p* = .001), ECOG performance status ≥ 2 (53.9% vs. 24.1%, *p* = .002) and severe ACE-27 score (43% vs. 26.7% in mild ACE-27 score, *p* = .014), see Supplementary Table 2. Next, we assessed prediction rates of relevant GAS in combination with geriatric age (cut-off age ≥ 80 vs. <80 years) for postoperative morbidity and mortality (Table 2). On multivariate logistic regression analysis, postoperative CDC grade ≥ IIIb was significantly predicted by sFI ≥ 2 (OR: 2.06, 95% CI: 1.27–3.34, *p* = .004), ECOG performance status ≥ 2 (OR: 2.78, 95% CI: 1.18–6.54, *p* = .019) and severe ACE-27 score (OR: 2.07, 95% CI: 1.13–3.79, *p* = .019). Eventually, we assessed 90-d mortality rate prediction of GAS in combination with geriatric age (Supplementary Table 3). On multivariate logistic regression analysis, 90-d mortality was significantly associated with lower BMI (OR: 0.89, 95% CI: 0.81–0.99, *p* = .024), ECOG performance status ≥ 2 (OR: 2.87, 95% CI: 1.18–7.96, *p* = .04) and, in particular, CDC grade ≥ IIIb (OR: 22.91, 95% CI: 8.74–60.09, *p* < .001; Supplementary Table 3).

## Discussion

In the present study, we report high 30-d severe complications rates with CDC grade ≥ IIIb of 41.2% and 90-d mortality of 26.9% after RC in patients age ≥ 80 years. Our meticulous analysis of validated GAS revealed sFI ≥ 2, ECOG performance status ≥ 2 and severe ACE-27 score as best GAS for predicting 30-d morbidity (= CDC grade ≥ IIIb). Consequently, our results highlight the strong association between patient age, frailty and postoperative morbidity and mortality after RC [15, 16, 22, 23]. Hence, these data confirm that prior subjective assessment based on age and BMI can no longer be mainstay in clinical practice. Finally, this study provides further information about the usefulness of additional GAS and classification of patients when undergoing RC. We present relevant predictive capacity of standard GAS to predict complications as well as mortality after RC. The demographic change as well as the improved health care systems and associated physical performance status of patients represent an increasing challenge in the application of rigid age limits for clinical decision making for RC [12, 24]. Along this line we decided to define the geriatric patient population as being ≥ 80 years of age as it represents the current cut-off, at which real-life clinical treatment decisions are becoming increasingly difficult when assessing patients to be suitable for RC.

Our data clearly emphasizes the critical view on RC in patients ≥ 80 years of age due to a high 90-d mortality rate of 26.9% compared to 4.8% in patients < 80 years of age in a recent study cohort from 2016–2021. These circumstances are of great importance because the number of patients age ≥ 80 years significantly increased in our department from 1.23% between 1968 and 1990 towards 17.12% between 2016 and 2022 (*p* < .001), which is comparable to international population based studies [2, 6, 24, 25]. Our study presents different results than the multi-center evaluation of 1955 patients (*n* = 251 (13%) were ≥ 80-year-old) undergoing RC between 2006 and 2021 with no differences in any grades of postoperative CDC [24]. However, our study underlines several previous associations between increased complications and overall mortality once patients ≥ 80 years of age suffered 41.1% severe CDC grade ≥ IIIb compared to 27.8% in patients < 80 years of age [8]. Our logistic regression analysis revealed 90-d mortality to be mainly influenced by presence of CDC grade ≥ IIIb (OR: 22.91, 95% CI: 8.74–60.09, *p* < .001), thus by severe complication rates, in combination with patients age ≥ 80 years (OR: 7.84, 95% CI: 3.28–18.74, *p* < .001). Hence, we supposed the necessity to use additional GAS for individual treatment approaches. Here, our data reveal sFI ≥ 2 (OR: 2.06, 95% CI: 1.27–3.34, *p* = .004), severe ACE-27 score (OR: 2.07, 95% CI: 1.13–3.79, *p* = .019) and ECOG performance status ≥ 2 (OR: 2.78, 95% CI: 1.18–6.54, *p* = .019) as best suited GAS to predict CDC grade ≥ IIIb, while 90-d mortality was most significantly predicted by CDC grade ≥ IIIb (OR: 22.91, 95% CI: 8.74–60.09, *p* < .001) and ECOG ≥ 2 (OR: 2.87, 95% CI: 1.05–7.86, *p* = .04). Interestingly, next to CDC grade ≥ IIIb and ECOG status ≥ 2 (OR: 2.87, 95% CI: 1.05–7.86, *p* = .04), 90-d mortality was also associated with lower BMI (OR: 0.89, 95% CI: 0.81–0.99, *p* = .024). These observations are comparable with previous studies on the ”obesity paradox”, according to which obesity appears to protect against short-term mortality [26].

Our 90-d mortality rate in patients aged ≥ 80 years differs from previous studies [27]. Either due to missing sub-analyses for the older age or due to lower cut-off values such as 65, 70 or 75 years [6, 15, 16, 28]. Regarding the rapidly changing trends in elderly patients and better physical health status with indication for RC, we proclaim to adapt these upper limits of patients age for decision making process to 80 years [2, 25]. Mayr et al. published a similar evaluation of the ACE-27, the CCI, ECOG status and ASA score in a cohort of 555 patients undergoing RC from 2000 to 2010 [22]. They concluded that each of the four GAS was able to significantly predict 90-d overall mortality (7.9%), while ACE-27 (+ 29.8%, OR: 1.72, *p* = .004, area under the curve (AUC): 73.8) revealed best predictive capabilities additional to defined clinical variables like age and clinical distant metastatic tumour stage for linear regression model. Interestingly, ECOG performance status improved their model with lowest impact (+ 13.5%, OR: 1.61, *p* = .036, AUC: 74.7) which differs from our results. However, ASA score revealed similar results like ACE-27 (+ 28.3%, OR: 2.19, *p* = .004, AUC 76.1). These observations are in line with Schulz et al. who published a study cohort of 1206 patients undergoing RC from 2004 to 2010 [15]. They reported 90-d mortality (7.6% vs. 3.2%) and CDC grade ≥ 3 (14.6% vs. 9.4%) to be about twice as high in patients with ASA ≥ 3 compared to ASA < 3. However, they did not investigate any further GAS. Furthermore, Sathianathen et al. presented the newly established sFI, which we also used in our study, and compared its prediction rates on 30-d morbidity within a database of 5516 patients [16]. They described the sFI (≥ 3) to outperform ASA score in predicting CDC grade ≥ 3 (OR: 3.22, 95% CI: 2.01–5.17), which was consistent in their subgroup of patients aged ≥ 65 years. Finally, Boorjian et al. published long-term follow up data of 891 patients undergoing RC between 1994 and 2005 [23]. They evaluated CCI, ASA score and ECOG performance status and concluded CCI (HR 1.23, *p* < .0001) as well as ECOG performance status (HR: 1.97, *p* < .0001) as independent predictors of 5-year all-cause mortality. On the one hand, all reported studies demonstrate the heterogeneity of GAS in predicting morbidity and mortality after RC which highlights the novelty and importance of our study in comparison of eight GAS. On the other hand, they strengthen the need of prospectively validated GAS to achieve higher level of evidence which reflects the comparatively low available data in current EAU guidelines [12].

There are limitations to this study that need to be considered. Next to the retrospective design, we calculated retrospectively the following GAS: CCI, ACE-*27* score, POSPOM score, ISAR Screening and the sFI. Even though each GAS was carried out with the greatest accuracy and internal control, the results need to be evaluated prospectively in further studies. The challenge in clinical implementation lies in the work-intensive and time-consuming calculation of all GAS. Moreover, results of the octogenarian population are generally limited due to their smaller numbers within the study population (15.8%). Furthermore, our findings might be difficult to compare with institutions with higher frequencies of neoadjuvant chemotherapy and robotic-assisted surgery. Despite comprehensive available clinicopathological and perioperative patient characteristics, it was not possible to identify social and environmental factors that might also influence morbidity and mortality outcomes [29]. Notably, we did not stratify outcomes by surgeon volume once best evidence relies more on the hospital volume, which defines our institution with approximately > 70 RC per year as a high-volume center [11].

## Conclusion

In summary, we derive two recommendations for RC from our results. First, patients age ≥ 80 years need to be informed about 90-d mortality rates up to 26.9% which is significantly higher than previous data of overall patients’ cohorts between 5.7% and 9.0% [22, 27]. Second, we consider CDC ≥ IIIb rates of 41.7% in patients age ≥ 80 years as unacceptable standard of care in an ageing RC population. Therefore, we strongly suggest to use the predicting factors sFI score > 2, ECOG performance status > = 2 and severe ACE-27 score to counsel patients either in favor for RC if they are below these cut-off values, or rather for a bladder-sparing multimodal treatment concept if above this cut-off [30]. Finally, these results need to be evaluated in prospective trials.

## Electronic supplementary material

Below is the link to the electronic supplementary material.

Supplementary Material 1

Supplementary Material 2

Supplementary Material 3

## Data Availability

The data that support the findings of this study are available from the corresponding author upon reasonable request.
