# Supplementary material for: Radical cystectomy in patients aged < 80 years versus ≥ 80 years: analysis of preoperative geriatric assessment scores in predicting postoperative morbidity and mortality
Source: World J Urol. 2024 Sep 30;42(1):552. doi: 10.1007/s00345-024-05248-y (PMC11442567; doi:10.1007/s00345-024-05248-y)
Supplement: Supplementary file 1 — Supplementary Material 1 [file 345_2024_5248_MOESM1_ESM.docx]

| **Supplementary Table 1. Patients' baseline characteristics, n= 424**   \| **Patients** \| **Mean (SD)** \| **Median (IQR)** \| **n=** \| \| --- \| --- \| --- \| --- \| \|  \|  \|  \|  \| \| Age at RC \| 69.75 (9.75) \| 71.00 (68.82;70.68) \| 424 \| \| BMI \| 27.71 (5.38) \| 26.80 (25.11;30.30) \| 424 \| \| Intraoperative blood loss (ml) \| 1,000.06 (0.856) \|  \| 339 \| \| Operating time (minutes) \| 362.67 (87.18) \| 362.00 (301.00;415.98) \| 423 \| \| Days ICU postoperative \| 2.73 (6.54) \| 1.00 (0.59;11.73) \| 409 \| \|  \|  \|  \|  \| \| Geriatric Assesments Scores \|  \|  \|  \| \| Charlson Comorbidity Index \| 5.89 (2.64) \| 6.00 (4.62;7.17) \| 424 \| \| ACE-27 \| 2.05 (0.97) \| 2.00 (1.58;2.52) \| 424 \| \| POSPOM \| 31.47 (4.33) \| 30.00 (29.39;33.56) \| 424 \| \| Barthel-Index (calculated) \| 99.21 (2.51) \| 100.00 (98.00;100.42) \| 310 \| \| ISAR-Screening \| 1.68 (0.75) \| 2.00 (1.32;2.05) \| 424 \| \| Simplified Frailty Index \| 2.05 (1.18) \| 2.00 (1.45;2.62) \| 424 \| \| ECOG Performance Status (prior RC) \| 0.68 (0.67) \| 1.00 (0.36;1.01) \| 208 \| \| ASA Risk Stratification \| 2.79 (0.63) \| 3.00 (2.49;3.09) \| 415 \| \|  \|  \|  \|  \| \|  \| **n=424 (%)** \|  \|  \| \| Male patients \| 328 (77.4) \|  \|  \| \| Female patients \| 96 (22.6) \|  \|  \| \| Patients age ≥80 \| 67 (15.8) \|  \|  \| \| Patients age <80 \| 357 (84.2) \|  \|  \| \| Neoadjuvant chemotherapy \| 29 (6.8) \|  \|  \| \| Open-surgery \| 380 (89.6) \|  \|  \| \| Robotic-assisted surgery \| 44 (10.4) \|  \|  \| \|  \|  \|  \|  \| \| RC Indication \|  \|  \|  \| \| Curative (bladder cancer) \| 396 (93.4) \|  \|  \| \| Other (palliative, non-oncological) \| 28 (6.6) \|  \|  \| \|  \|  \|  \|  \| \| Urinary Diversion \|  \|  \|  \| \| Incontinent urinary diversion \| 352 (83.0) \|  \|  \| \| Continent urinary diversion \| 66 (15.6) \|  \|  \| \| None \| 6 (1.4) \|  \|  \| \|  \|  \|  \|  \| \| Last TURBT T-Stages (prior RC) \|  \|  \|  \| \| T0 \| 1 (0.24) \|  \|  \| \| Tis/Ta \| 48 (11.32) \|  \|  \| \| T1 \| 105 (24.76) \|  \|  \| \| T2 \| 209 (49.29) \|  \|  \| \| T4 \| 2 (0.47) \|  \|  \| \| Unknown \| 59 (13.92) \|  \|  \| \|  \|  \|  \|  \| \| Final Pathology \|  \|  \|  \| \| Urothelial Cancer \| 365 (86.1) \|  \|  \| \| Squamous Cell Cancer \| 12 (2.83) \|  \|  \| \| Othel Histology \| 29 (6.8) \|  \|  \| \| No cancer \| 18 (4.2) \|  \|  \| \|  \|  \|  \|  \| \| RC T-Stages \|  \|  \|  \| \| T0 \| 36 (8.49) \|  \|  \| \| Tcis/Ta \| 37 (8.73) \|  \|  \| \| T1 \| 48 (11.32) \|  \|  \| \| T2 \| 85 (20.05) \|  \|  \| \| T3 \| 128 (30.18) \|  \|  \| \| T4 \| 47 (11.09) \|  \|  \| \| Unknown \| 43 (10.14) \|  \|  \| \|  \|  \|  \|  \| \| RC N-Stages \|  \|  \|  \| \| N0 \| 250 (58.96) \|  \|  \| \| N1 \| 37 (8.73) \|  \|  \| \| N2 \| 41 (9.67) \|  \|  \| \| N3 \| 13 (3.07) \|  \|  \| \| NX \| 31 (7.31) \|  \|  \| \| Unknown \| 52 (12.26) \|  \|  \| \|  \|  \|  \|  \| \| RC R-Stages \|  \|  \|  \| \| R0 \| 315 (74.29) \|  \|  \| \| R1 \| 39 (9.2) \|  \|  \| \| R2 \| 2 (0.47) \|  \|  \| \| RX \| 8 (1.89) \|  \|  \| \| Unknown \| 60 (14.2) \|  \|  \| \|  \|  \|  \|  \| \| Clavien-Dindo Classification \|  \|  \|  \| \| Grade I \| 26 (6.13) \|  \|  \| \| Grade II \| 173 (40.80) \|  \|  \| \| Grade III \| 77 (18.16) \|  \|  \| \| Grade IV \| 37 (8.73) \|  \|  \| \| Grade V \| 17 (4.01) \|  \|  \| \| None \| 94 (22.17) \|  \|  \| \|  \|  \|  \|  \| \| Postoperative Outcome \|  \|  \|  \| \| 0-1 complications 30d after RC \| 200 (47.17) \|  \|  \| \| ≥2 complications 30d after RC \| 224 (52.83) \|  \|  \| \| Revisions Surgery 60d after RC \| 58 (13.68) \|  \|  \| \| Death 30d after RC \| 17 (4.01) \|  \|  \| \| Death 90d after RC \| 35 (8.25) \|  \|  \| \|  \|  \|  \|  \| \| ASA Risk Stratification I \| 1 (0.2) \|  \|  \| \| ASA Risk Stratification II \| 164 (38.7) \|  \|  \| \| ASA Risk Stratification III \| 222 (52.4) \|  \|  \| \| ASA Risk Stratification Iv \| 28 (6.6) \|  \|  \| \|  \|  \|  \|  \| \| Preoperative intake of NOAC \| 31 (7.3) \|  \|  \| \| Preoperative intake of acetylsalicylic acid \| 125 (29.5) \|  \|  \| \| Preoperative intake of coumarin \| 15 (3.5) \|  \|  \| \|  \|  \|  \|  \| \| Preoperative Comorbidities \|  \|  \|  \| \| Secondary malignant neoplasms \| 173 (40.8) \|  \|  \| \| Nicotine abuse \| 124 (29.2) \|  \|  \| \| Diabetes mellitus \| 86 (20.3) \|  \|  \| \| Ischemic heart disease \| 71 (16.7) \|  \|  \| \| Chronic heart failure \| 32 (7.5) \|  \|  \| \| Peripheral arterial occlusion disease \| 39 (9.2) \|  \|  \| \| Chronic obstructive pulmonary disease \| 97 (22.9) \|  \|  \| \| Chronic respiratory insufficiency \| 11 (2.6) \|  \|  \| \| Moderate or severe liver disease \| 3 (0.7) \|  \|  \| |
| --- | --- | --- | --- | --- | --- | --- | --- | --- | --- | --- | --- | --- | --- | --- | --- | --- | --- | --- | --- | --- | --- | --- | --- | --- | --- | --- | --- | --- | --- | --- | --- | --- | --- | --- | --- | --- | --- | --- | --- | --- | --- | --- | --- | --- | --- | --- | --- | --- | --- | --- | --- | --- | --- | --- | --- | --- | --- | --- | --- | --- | --- | --- | --- | --- | --- | --- | --- | --- | --- | --- | --- | --- | --- | --- | --- | --- | --- | --- | --- | --- | --- | --- | --- | --- | --- | --- | --- | --- | --- | --- | --- | --- | --- | --- | --- | --- | --- | --- | --- | --- | --- | --- | --- | --- | --- | --- | --- | --- | --- | --- | --- | --- | --- | --- | --- | --- | --- | --- | --- | --- | --- | --- | --- | --- | --- | --- | --- | --- | --- | --- | --- | --- | --- | --- | --- | --- | --- | --- | --- | --- | --- | --- | --- | --- | --- | --- | --- | --- | --- | --- | --- | --- | --- | --- | --- | --- | --- | --- | --- | --- | --- | --- | --- | --- | --- | --- | --- | --- | --- | --- | --- | --- | --- | --- | --- | --- | --- | --- | --- | --- | --- | --- | --- | --- | --- | --- | --- | --- | --- | --- | --- | --- | --- | --- | --- | --- | --- | --- | --- | --- | --- | --- | --- | --- | --- | --- | --- | --- | --- | --- | --- | --- | --- | --- | --- | --- | --- | --- | --- | --- | --- | --- | --- | --- | --- | --- | --- | --- | --- | --- | --- | --- | --- | --- | --- | --- | --- | --- | --- | --- | --- | --- | --- | --- | --- | --- | --- | --- | --- | --- | --- | --- | --- | --- | --- | --- | --- | --- | --- | --- | --- | --- | --- | --- | --- | --- | --- | --- | --- | --- | --- | --- | --- | --- | --- | --- | --- | --- | --- | --- | --- | --- | --- | --- | --- | --- | --- | --- | --- | --- | --- | --- | --- | --- | --- | --- | --- | --- | --- | --- | --- | --- | --- | --- | --- | --- | --- | --- | --- | --- | --- | --- | --- | --- | --- | --- | --- | --- | --- | --- | --- | --- | --- | --- | --- | --- | --- | --- | --- | --- | --- | --- | --- | --- | --- | --- | --- | --- | --- | --- | --- | --- | --- | --- | --- | --- | --- | --- | --- | --- | --- | --- | --- | --- | --- | --- | --- | --- | --- | --- | --- | --- | --- | --- | --- | --- | --- | --- | --- | --- | --- | --- | --- | --- | --- | --- | --- | --- | --- | --- | --- | --- | --- | --- | --- | --- | --- | --- | --- | --- | --- | --- | --- | --- | --- | --- | --- | --- | --- | --- | --- | --- | --- | --- | --- | --- | --- | --- | --- | --- | --- | --- | --- | --- | --- | --- | --- | --- | --- | --- | --- | --- | --- | --- | --- | --- | --- | --- | --- | --- | --- | --- |

Abbreviations: SD: standard deviation, IQR: interquartile range, RC: radical cystectomy, BMI: body mass index, ICU: intensive care unit, ACE 27: Adult Comorbidity Evaluation 27, POSPOM: Preoperative Score to Predict Postoperative Mortality, ISAR: Identification of Seniors at Risk, ECOG: Eastern Cooperative Oncology Group, ASA: American Society of Anaesthesiologists risk stratification, NOAC: new oral anticoagulants
