# Supplementary material for: Radical cystectomy in patients aged < 80 years versus ≥ 80 years: analysis of preoperative geriatric assessment scores in predicting postoperative morbidity and mortality
Source: World J Urol. 2024 Sep 30;42(1):552. doi: 10.1007/s00345-024-05248-y (PMC11442567; doi:10.1007/s00345-024-05248-y)
Supplement: Supplementary file 2 — Supplementary Material 2 [file 345_2024_5248_MOESM2_ESM.docx]

**Supplementary Table 2. Correlation of patient characteristics and postoperative Clavien-Dindo Complications < grade IIIb versus ≥ grade IIIb**

| **Characteristic** | **Maximum CDC <IIIb per patient (%)** | **Maximum CDC ≥IIIb per patient (%)** | **p-value** |
| --- | --- | --- | --- |
| **Patient Age** |  |  | 0.047* |
| < 80 (%) | 198 (85.71) | 76 (76.77) |  |
| ≥ 80 (%) | 33 (14.29) | 23 (23.23) |  |
| **BMI** |  |  | 0.279 |
| < 30 (%) | 185 (80.09) | 74 (74.75) |  |
| ≥ 30 (%) | 46 (19.91) | 25 (25.25) |  |
| **ECOG Performance Status** |  |  | 0.002* |
| < 2 | 101 (89.38) | 32 (69.57) |  |
| ≥ 2 | 12 (10.62) | 14 (30.43) |  |
| **ISAR-Screening** |  |  | 0.206 |
| Mean (SD) | 1.71 (1.04) | 1.82 (0.98) |  |
| **ASA Classification** |  |  | 0.199 |
| < 3 | 86 (37.89) | 28 (28.87) |  |
| ≥ 3 | 141 (62.11) | 69 (71.13) |  |
| **Simplified Frailty Index** |  |  | 0.002* |
| < 2 | 158 (68.4) | 50 (50.51) |  |
| ≥ 2 | 73 (31.6) | 49 (49.49) |  |
| **POSPOM** |  |  | 0.011* |
| < 27 | 107 (46.32) | 31 (31.31) |  |
| ≥ 28 | 124 (53.68) | 68 (68.69) |  |
| **ACE-27 Score** |  |  | 0.014* |
| None | 25 (10.82) | 8 (8.08 |  |
| Mild | 75 (32.47) | 27 (27.27) |  |
| Moderate | 78 (33.77) | 24 (24.24) |  |
| Severe | 53 (22.94) | 40 (40.4) |  |
| **Charlson Comorbidity Index** |  |  | 0.001* |
| 1-2 | 71 (30.74) | 18 (18.18) |  |
| 3-4 | 67 (29) | 18 (18.18) |  |
| ≥ 5 | 93 (40.26) | 63 (63.64) |  |
| **Neoadjuvant Chemotherapy** |  |  | 0.496 |
| None | 212 (91.77) | 93 (93.94) |  |
| Yes | 19 (8.23) | 6 (6.06) |  |
| **RC Indication** |  |  | 0.169 |
| Curative (bladder cancer) | 221 (95.67) | 91 (91.92) |  |
| Other (palliative, non-oncological) | 10 (4.33) | 8 (8.08) |  |
| **Robotic-assisted RC** |  |  | 0.143 |
| None | 208 (90.04) | 94 (94.95) |  |
| Yes | 23 (9.96) | 5 (5.05) |  |

Abbreviations: *statistically significant results, BMI: Body Mass Index, ECOG: Eastern Cooperative Oncology Group, ISAR: Identification of Seniors at Risk, ASA: American Society of Anaesthesiologists risk stratification, POSPOM: Preoperative Score to Predict Postoperative Mortality, ACE 27: Adult Comorbidity Evaluation 27, RC: Radical Cystectomy
