# Supplementary material for: Radical cystectomy in patients aged < 80 years versus ≥ 80 years: analysis of preoperative geriatric assessment scores in predicting postoperative morbidity and mortality
Source: World J Urol. 2024 Sep 30;42(1):552. doi: 10.1007/s00345-024-05248-y (PMC11442567; doi:10.1007/s00345-024-05248-y)
Supplement: Supplementary file 3 — Supplementary Material 3 [file 345_2024_5248_MOESM3_ESM.docx]

**Supplementary Table 3: Binary logistic regression analysis** **of patients aged ≥ 80 years in addition to geriatric assessment scores to predict 90 days postoperative mortality**

| Groups | Age ≥ 80 + Scores | Multivariate Analysis | | | Univariate Analysis | | |
| --- | --- | --- | --- | --- | --- | --- | --- |
|  |  | **OR** | **95% CI** | **p value** | **OR** | **95% CI** | **p value** |
| 1 | **Age ≥ 80** | 6.61 | 3.17-13.79 | < .001* | 7.35 | 3.55-15.21 | < .001* |
|  | **BMI (metric)** | 0.89 | 0.81-0.99 | .024* | 0.88 | 0.81-0.96 | .006* |
|  |  |  |  |  |  |  |  |
| 2 | **Age ≥ 80** | 6.66 | 3.18-13.95 | < .001* | 7.35 | 3.55-15.21 | < .001* |
|  | ECOG < 2 (reference) |  |  |  |  |  |  |
|  | **ECOG ≥ 2** | 2.87 | 1.05-7.86 | .04* | 3.96 | 1.56-10,02 | .004* |
|  |  |  |  |  |  |  |  |
| 3 | **Age ≥ 80** | 7.01 | 3.37-14.57 | < .001* | 7.35 | 3.55-15.21 | < .001* |
|  | ASA < 2 (reference) |  |  |  |  |  |  |
|  | **ASA ≥ 2** | 1.51 | 0.69-3.34 | .304 | 1.81 | 0.85-3.87 | .126 |
|  |  |  |  |  |  |  |  |
| 4 | **Age ≥ 80** | 7.84 | 3.28-18.74 | < .001* | 7.35 | 3.55-15.21 | < .001* |
|  | CDC < IIIb (reference) |  |  |  |  |  |  |
|  | **CDC ≥ IIIb** | 22.91 | 8.74-60.09 | < .001* | 22.03 | 8.81-55.07 | < .001* |
|  |  |  |  |  |  |  |  |
| 5 | **Age ≥ 80** | 7.33 | 3.53-15.24 | < .001* | 7.35 | 3.55-15.21 | < .001* |
|  | sFI < 2 (reference) |  |  |  |  |  |  |
|  | **sFI ≥ 2** | 1.02 | 0.48-2.15 | .96 | 1.21 | 0.6-2.46 | .593 |
|  |  |  |  |  |  |  |  |
| 6 | **Age ≥ 80** | 5.84 | 2.53-13.49 | < .001* | 7.35 | 3.55-15.21 | < .001* |
|  | POSPOM < 28 (reference) |  |  |  |  |  |  |
|  | **POSPOM ≥ 28** | 1.63 | 0.6-4.37 | .335 | 3.48 | 1.48-8.16 | .004* |
|  |  |  |  |  |  |  |  |
| 7 | **Age ≥ 80** | 6.91 | 3.32-14.39 | < .001* | 7.35 | 3.55-15.21 | < .001* |
|  | ACE 27 mild (reference) |  |  |  |  |  |  |
|  | ACE27 moderate | 1.39 | 0.54-3.54 | 1.39 | 1.34 | 0.55-3.31 | .519 |
|  | **ACE 27 severe** | 1.78 | 0.7-4.52 | .225 | 1.85 | 0.76-4.52 | .174 |

Abbreviations: OR: Odds ratio (β), CI: confidence interval, *statistically significant results, BMI: Body Mass Index, ECOG: Eastern Cooperative Oncology Group, ASA: American Society of Anaesthesiologists risk stratification, CDC: Clavien-Dindo Classifications (30 days postoperative complications), sFI: Simplified Frailty Index, POSPOM: Preoperative Score to Predict Postoperative Mortality, ACE 27: Adult Comorbidity Evaluation 27
